# Real‐time deep artifact suppression using recurrent U‐Nets for low‐latency cardiac MRI

**DOI:** 10.1002/mrm.28834

**Published:** 2021-05-25

**Authors:** Olivier Jaubert, Javier Montalt‐Tordera, Dan Knight, Gerry J. Coghlan, Simon Arridge, Jennifer A. Steeden, Vivek Muthurangu

**Affiliations:** ^1^ Department of Computer Science University College London London United Kingdom; ^2^ UCL Centre for Translational Cardiovascular Imaging University College London London United Kingdom; ^3^ Department of Cardiology Royal Free London NHS Foundation Trust London United Kingdom

**Keywords:** cardiac MRI, deep learning, image reconstruction, interventional, real‐time

## Abstract

**Purpose:**

Real‐time low latency MRI is performed to guide various cardiac interventions. Real‐time acquisitions often require iterative image reconstruction strategies, which lead to long reconstruction times. In this study, we aim to reconstruct highly undersampled radial real‐time data with low latency using deep learning.

**Methods:**

A 2D U‐Net with convolutional long short‐term memory layers is proposed to exploit spatial and preceding temporal information to reconstruct highly accelerated tiny golden radial data with low latency. The network was trained using a dataset of breath‐hold CINE data (including 770 time series from 7 different orientations). Synthetic paired data were created by retrospectively undersampling the magnitude images, and the network was trained to recover the target images. In the spirit of interventional imaging, the network was trained and tested for varying acceleration rates and orientations. Data were prospectively acquired and reconstructed in real time in 1 healthy subject interactively and in 3 patients who underwent catheterization. Images were visually compared to sliding window and compressed sensing reconstructions and a conventional Cartesian real‐time sequence.

**Results:**

The proposed network generalized well to different acceleration rates and unseen orientations for all considered metrics in simulated data (less than 4% reduction in structural similarity index compared to similar acceleration and orientation‐specific networks).

The proposed reconstruction was demonstrated interactively, successfully depicting catheters in vivo with low latency (39 ms, including 19 ms for deep artifact suppression) and an image quality comparing favorably to other reconstructions.

**Conclusion:**

Deep artifact suppression was successfully demonstrated in the time‐critical application of non‐Cartesian real‐time interventional cardiac MR.

## 1 INTRODUCTION

MR‐guided cardiac catheterization is a growing field that has promising applications for right heart catheterization, ablation therapy, endomyocardial biopsies, and stent positioning.^1^ MR catheter guidance relies on real‐time interactive imaging^2^ that is now standard on most commercial systems. However, commercially available sequences are often limited to moderately accelerated Cartesian acquisitions with relatively low spatial and temporal resolutions.

Developments in iterative reconstruction and non‐Cartesian sampling have shown the possibility to further accelerate imaging. Iterative reconstruction techniques (including compressed sensing (CS)^3^), combined with non‐Cartesian undersampling, have been used for retrospective reconstruction of high spatiotemporal resolution real‐time MR acquisitions.^4^ However, these methods are challenging in time‐critical applications such as interventional MRI. Nevertheless, low latency non‐Cartesian frameworks have been proposed for both noniterative (radial GRAPPA^5^) and iterative reconstructions.^6^, ^7^, ^8^, ^9^ Indeed, iterative methods have been successfully applied to catheter guidance.^8^ The main problem with these approaches is the need for high‐end computer hardware, often relying on multi‐GPU systems to reconstruct images with low latency.

Recently, it has been shown that deep learning (DL) methods are able to reconstruct highly undersampled MR images^10^ in many different applications, including cardiac 2D CINE^11^, ^12^, ^13^ and real‐time cardiac MRI.^14^ Several DL methods have been proposed, including end‐to‐end networks,^15^ unrolled networks with data consistency,^11^, ^16^, ^17^ and deep artifact suppression networks.^14^, ^18^ Importantly, reconstruction times using DL were significantly shorter than conventional iterative techniques, which is relevant when using less powerful computer hardware. This is especially true for the single‐pass deep artifact suppression techniques, opening up the possibility of DL reconstruction to catheter guidance.

In this study, we propose a framework that combines an accelerated interactive radial balanced steady‐state free precession (bSSFP) sequence with low‐latency machine‐learning deep artefact suppression. Our method utilizes a U‐Net^19^ with 2D convolutional long short‐term memory (ConvLSTM)^20^ blocks that exploit spatiotemporal redundancies. Importantly, our framework was set up to reconstruct images on the scanner, enabling use during MR‐guided cardiac catheterization. The main aims of this study were: 1) to demonstrate the feasibility of using our DL framework to reconstruct high‐quality real‐time images with low latency (< 200 ms),^1^ 2) to assess whether the proposed DL method generalizes to different acceleration rates and unseen imaging planes, and 3) to assess whether the method provides adequate image quality for visual tracking of a device during cardiac catheterization.

## 2 METHODS

This study was approved by the local research ethics committee (ref. 19/LO/1561: site 1; 06/Q0508/124: site 2), and written consent was obtained in prospective and retrospective cohorts.

**FIGURE 1:**
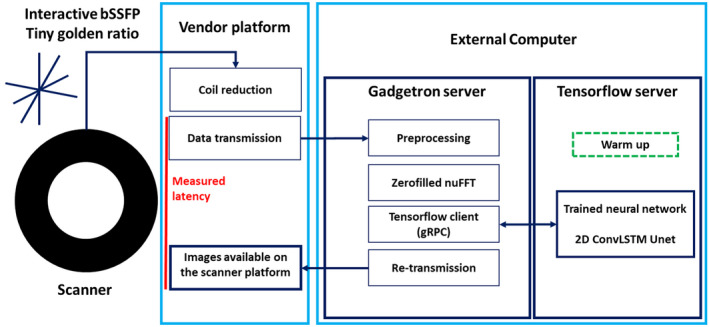
Proposed framework for real‐time deep artifact suppression. An interactive bSSFP acquisition continuously collects radial spokes incremented by the tiny golden angle. The data are sequentially coil‐compressed, transmitted to the external computer, preprocessed, gridded, and artifact‐suppressed using the proposed network on a local (warmed‐up) server before being sent back to the scanner for visualization. bSSFP, balanced steady‐state free precession; gRPC,remote procedure call

In summary, the proposed framework relies on a modified interactive tiny golden angle^21^, ^22^ radial bSSFP acquisition, an open‐source cross‐platform framework (Gadgetron, v4.1.1)^23^ for scanner integration, and a local server^24^ with a network ready for deep artefact suppression. The proposed framework is illustrated in Figure 1.

### 2.1 Training and test data

The training data for our DL model consisted of pairs of ground truth and synthetically undersampled (created from the ground truth data) magnitude cines (2D+time). The ground truth data were breath‐hold, retrospectively cardiac‐gated, Cartesian, bSSFP CINE data collected from 2 adult patient populations (site 1: adult heart disease patients, age: 56 ± 15 years old, heart rate: 76 ± 24 beat per min, 25 frames per cine, 385 CINEs; site 2: congenital heart disease patients, 34 ± 12 years old, heart rate: 69 ± 11 beats per min, 40 frames per cine, 385 CINEs) acquired at 2 clinical sites with different scanner models, Aera 1.5 Tesla (Siemens Healthineers) (site 1) and Avanto 1.5 Tesla (Siemens Healthineers) (site 2), from the same manufacturer (Siemens Healthineers, Erlangen, Germany). For both sites, the nominal sequence parameters were flip angle 58°, TR/TE 3.2/1.6 ms, matrix size 272 × 272, pixel size: 1.45 × 1.45 mm^2^, slice thickness: 10 mm, receiver bandwidth 793 Hz/pixel, and GRAPPA^25^ with acceleration factor of 2.

In total, 110 cines were collected in each of 7 orientations—short axis (SA or SAX), 4 chamber (FCH), left ventricular long axis (LVLA), right ventricular long axis (RVLA), left ventricular outflow tract (LVOT), right ventricular outflow tract (RVOT), and pulmonary artery (PA). This resulted in a total of 770 cines (magnitude only) that were spatially downsampled (bicubic interpolation) to the target pixel size used for prospective real time imaging (1.67 × 1.67 mm^2^) to create the appropriate target “ground truth” images.

To create the pairs of corrupted and ground truth images, the “ground truth” cine data were first Fourier‐transformed and gridded onto an undersampled tiny golden angle (~23.63°) radial trajectory. The undersampled data were then regridded and inverse Fourier‐transformed back into image space. Both the ground truth and artifact‐contaminated images were normalized to have signal intensities in the range [0, 1], and the center region was cropped to a 128 × 128 matrix to constrain the learning problem to the anatomy of interest (heart).

### 2.2 Architecture and training

**FIGURE 2:**
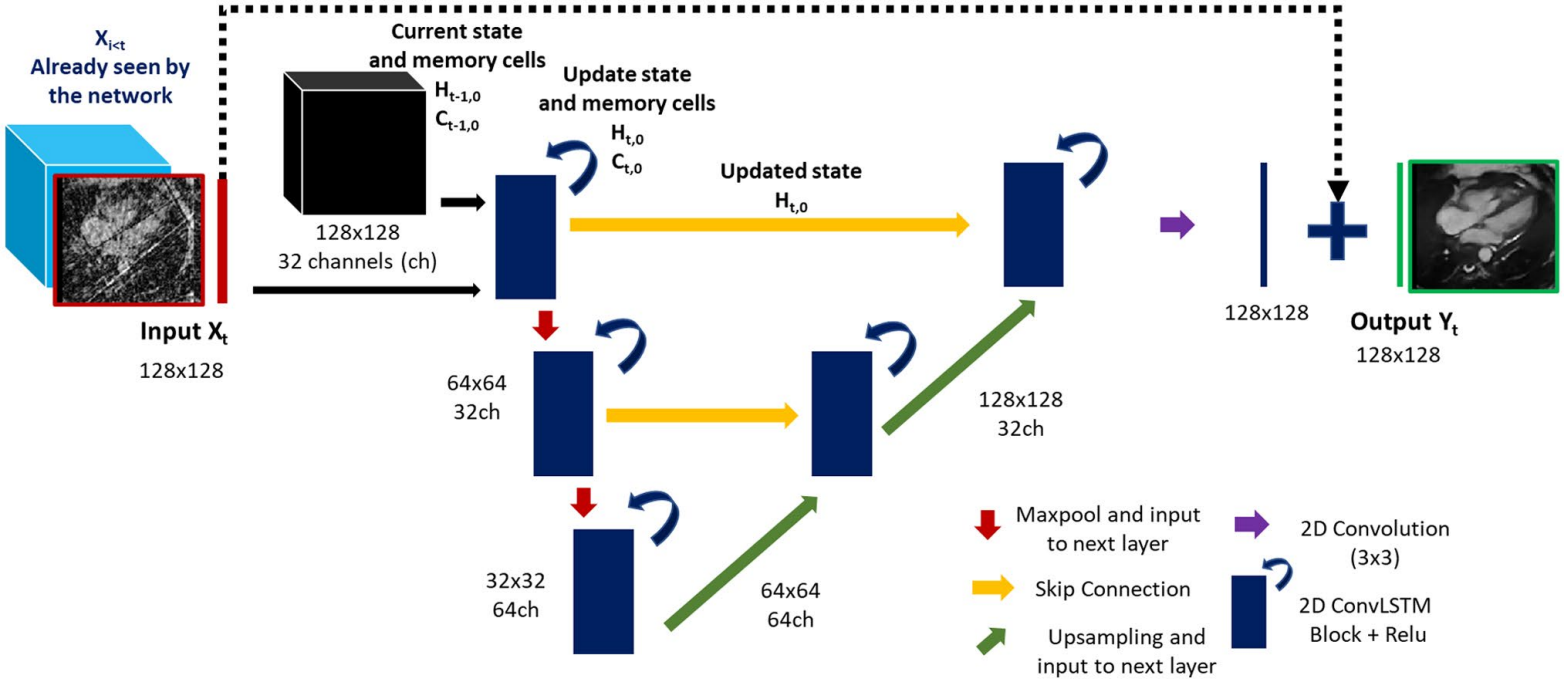
The proposed 2D residual U‐Net with multi‐scale operations, skip connections, and ConvLSTM layers to de‐alias the current image exploiting spatial and previous temporal information. Each ConvLSTM block has its own state (H_t−1_) and memory (C_t−1_). Following the input of the current 2D frame (X_t_), the states and memories are updated. The updated state (H_t_) is kept in memory but also forwarded to the following layer through skip connections, 2 × 2 max‐pooling, or 2 × 2 upsampling. ConvLSTM, convolutional long short‐term memory

The proposed deep artifact suppression network consists of a modified residual U‐Net, trained to recover the ground truth images from the corrupted input images. In this study, we replaced the 2D convolutional layers of the original U‐Net with 2D unidirectional ConvLSTM^20^ layers that reconstruct the current frame while retaining information about previous frames. 2D ConvLSTM are performed at each scale of the proposed U‐Net for both encoding and decoding blocks. A schematic of the proposed network is shown in Figure 2.

Parameters of the network included an input size of 128 × 128, 3 scales, and 32 filters learned per convolution (with 8 convolutions per ConvLSTM layer) at the first scale with a filter size of 3 × 3. A 2 × 2 max‐pool layer is performed within each encoding block and a 2 × 2 upsampling within each decoding block. The ConvLSTMs’ states (H_t_) and memory cells (C_t_) are updated as originally proposed.^20^

The network was implemented and trained using Python (v3.7.7) and TensorFlow (v2.2.0).^24^ Training was performed using 100 epochs, an initial learning rate of 0.001, batch size of 4, and an adaptive moment estimation algorithm (Adam).^26^ The data are split into time series of 8 frames at training (for memory considerations); however, the states and memory cells are reset only at the end of the complete time series. Training was performed on a Linux workstation (Ubuntu 18.08, Intel Core i9‐7900X, 3.3 GHz) using an NVIDIA Quadro GP100 (16 GB memory). All networks trained in this experiment had ~1.79 million trainable parameters and took 8 h and 40 min to train when training on the full dataset.

### 2.3 Model generalizability

We tested 3 aspects of model generalizability: variable acceleration, catheter visualization, and unseen orientations. Networks were compared using mean absolute error (MAE), structural similarity index (SSIM), mean squared error, and peak SNR ratio computed on frame 5 (when image quality stabilizes) to frame 25 of the test data set.

#### 2.3.1 Variable accelerations

**FIGURE 3:**
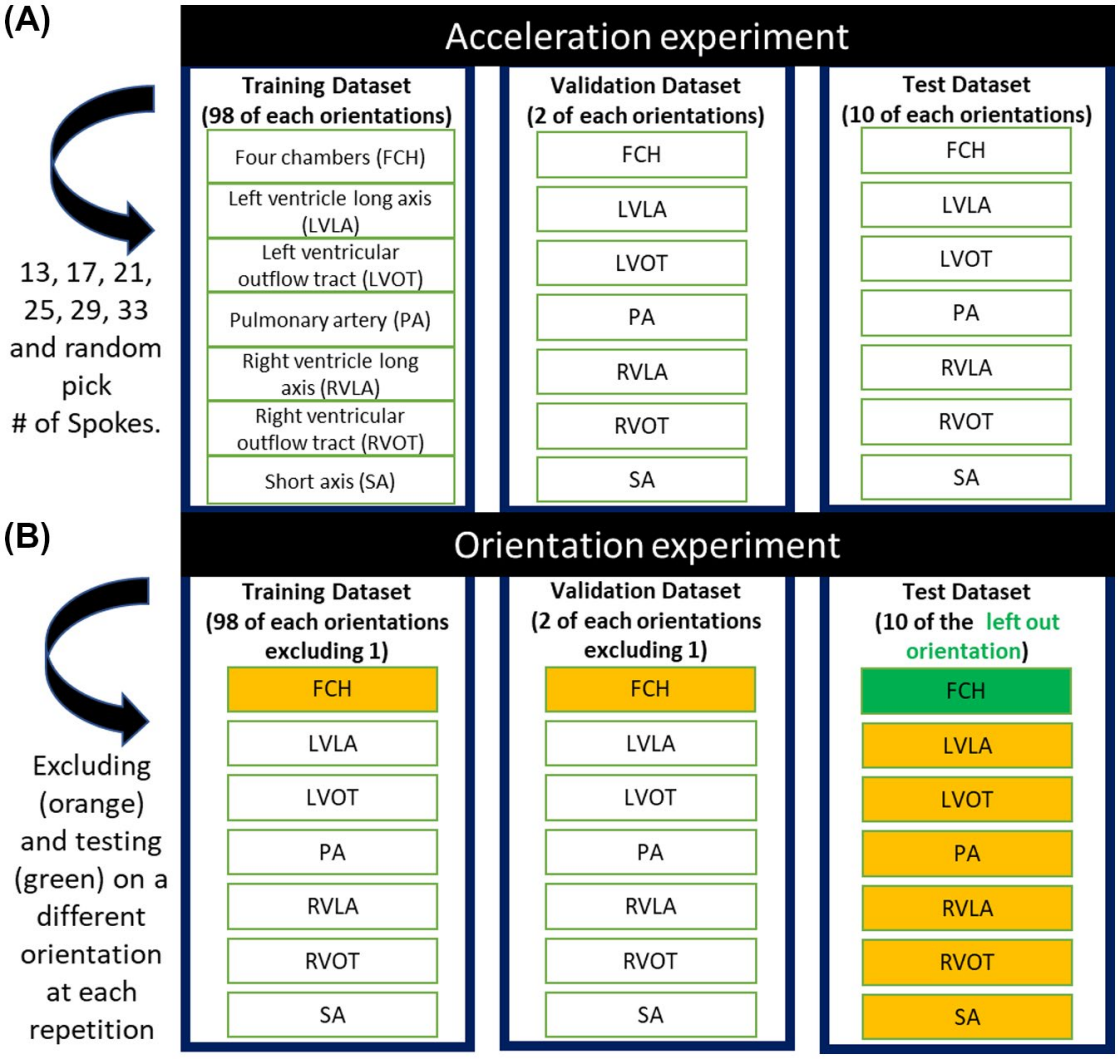
(A) Acceleration experiment. The dataset is split into 686 images for training, 14 for validation, and 70 for testing, with equal numbers for each orientation. Six “specific” networks are trained with 6 different undersampling rates. A seventh network (“generic”) is trained using varying acceleration rates randomly selected within the previously studied set. (B) Orientation experiment. Seven networks are trained, excluding 1 of the orientations in each. The resulting networks are tested on the left‐out orientation test data and compared to an eighth network, which is trained using all orientations to assess the generalizability of the network to unseen orientations

In this experiment, we assess whether a “generic” network trained on varying acceleration rates can provide satisfying image quality. Of the 110 cines per orientation in the dataset, 98 were used for training, 2 for validation, and 10 for testing, leading to 686 cines for training, 14 for validation, and 70 for testing. Input aliased images were simulated using [13, 17, 21, 25, 29, 33] spokes per frame, leading to acceleration factors of R = [23.2, 17.7, 14.4, 12.1, 10.4, 9.1] given 192 samples per readout.^27^, ^28^ A “specific” network was trained for each individual acceleration rate. A generic network was then trained with the same ground truth images but corrupted with acceleration rates randomly picked between those previously simulated, leading to approximately 114 training and 2 validation cines for each of the 6 acceleration factors considered (R = [23.2, 17.7, 14.4, 12.1, 10.4, 9.1]). The test set was reconstructed and assessed for each acceleration rate using both the “specific” and “generic” networks. The design of this experiment is summarized in Figure 3A.

#### 2.3.2 Catheter visualization

From the test set, 1 imaging series was modified to simulate a catheter balloon or guidewire, moving in and out of the imaging plane. The balloon was simulated using a single moving and vanishing 2D Gaussian with a maximal signal decrease of 80% in the blood pool. The guide wire was similarly simulated using 5 moving and vanishing 2D Gaussians. Simulations were performed with different acceleration rates to investigate the performance of the network when reconstructing moving structures not seen in the training dataset.

#### 2.3.3 Leave‐one‐out orientation experiment

In this experiment, we assess whether the proposed network is robust to “unseen” orientations (necessary for catheter guidance). To do this, we trained 7 networks using a leave‐one‐out (LOO) strategy for 1 of the orientations (ie, the network was trained using 6 of the 7 orientations: 588 images for training and 12 for validation) and tested on the left‐out orientation (ie, 10 images for testing). An eighth network was trained including all orientations (only 84 images per orientation for a total of 588 training images) for reference and compared to the LOO networks for each orientation. These networks were all trained with random undersampling similarly to the “generic” network. The design of this experiment is summarized in Figure 3B.

### 2.4 In vivo study

Prospective data were acquired at site 1 (Aera 1.5 Tesla, Siemens Healthineers) with an in‐house modified interactive radial bSSFP sequence with a tiny golden angle increment (as simulated in training). Acquisition parameters included flip angle = 58°, pixel size = 1.67 × 1.67 mm^2^, slice thickness = 10 mm, TR/TE = 3.2/1.55 ms, bandwidth = 793 Hz/pixel, FOV = 320 × 320 mm^2^, 17 spokes/frame (ie, ~54 ms/frame). Real‐time reconstructions were performed on a mid‐range GPU (GeForce GTX 1650 Ti, 4 GB memory, NVIDIA, Santa Clara, CA ) and laptop (Linux Ubuntu 18.08, Intel Core i7, 2.60 GHz, 8 GB RAM). On‐site reconstructions were performed using the Gadgetron framework and TensorFlow Serving API. During acquisition, the data are sequentially coil‐compressed (8 virtual coils), transferred to the external computer, preprocessed, gridded, coil‐combined (sum of squares), deep artifact‐suppressed, transferred back to the vendor platform, and displayed (Figure 1). More details on the implementation can be found in Supporting Information Text S1. To reduce initial latency due to “lazy” initialization of the network, the served network was warmed up using 2 frames with random Gaussian noise at startup. The effect of warmup on the image SSIM of initial frames is shown in Supporting Information Figure S1.

A healthy subject was acquired for: 1) the assessment of timings and latency (measured between the data transmission of the last spoke from the vendor platform and the moment the image is ready for display on the vendor platform (Figure 1), and 2) the qualitative assessment of the framework’s robustness to changes in FOV (acquired FOVs of 320 × 320, 360 × 360, 420 × 420 mm^2^) and imaging resolution (acquired resolutions of 1.5 × 1.5, 1.67 × 1.67, 2 × 2 mm^2^). In addition, data were acquired in 3 adult patients during right heart catheterization (2 patients with pulmonary hypertension associated with connective tissue disease and 1 patient with pulmonary hypertension associated with congenital heart disease) to assess cardiac, vascular, and catheter visualization. Data were reconstructed in real time using 17 spokes per frame (54 ms) and were also retrospectively reconstructed with 13, 21, 25, 29, and 33 spokes per frame—leading to temporal resolutions of 41, 67, 80, 93, and 106 ms.

In the patients, our new sequence was visually compared to conventional real‐time Cartesian Cardiac MR (Cartesian bSSFP, flip angle = 50°, pixel size = 2.5 × 2.5 mm^2^, slice thickness = 10 mm, TR/TE = 3.2/1.55 ms, bandwidth = 793 Hz/pixel, FOV = 320 × 320 mm^2^, 126 ms/frame), as well as a sliding window and CS reconstruction of the radial raw data. The sliding window reconstruction^21^ used a fixed step size of 17 spokes and a window width of 99 spokes (centered on the same k‐space line as the gridded reconstruction). The CS reconstruction was performed using the Berkeley Advanced Reconstruction Toolbox^29^ with temporal total variation regularization.^4^ Coil sensitivities were determined using ESPIRIT^30^; there were 50 conjugate gradient iterations and 5 iterations using alternating method of multipliers,^31^ with a regularization strength of λ = 0.01. For memory considerations, the CS time‐series were reconstructed in blocks of 20 frames.

### 2.5 Statistics

Multiple distributions in the test set metrics appeared non‐normal when tested for normality using Shapiro‐Wilk test. Therefore, paired Wilcoxon signed rank tests were performed to assess the statistical significance of the differences between “generic” and “specific” networks as well as between “seen” and “unseen” orientation networks.

## 3 RESULTS

### 3.1 Variable acceleration

**FIGURE 4:**
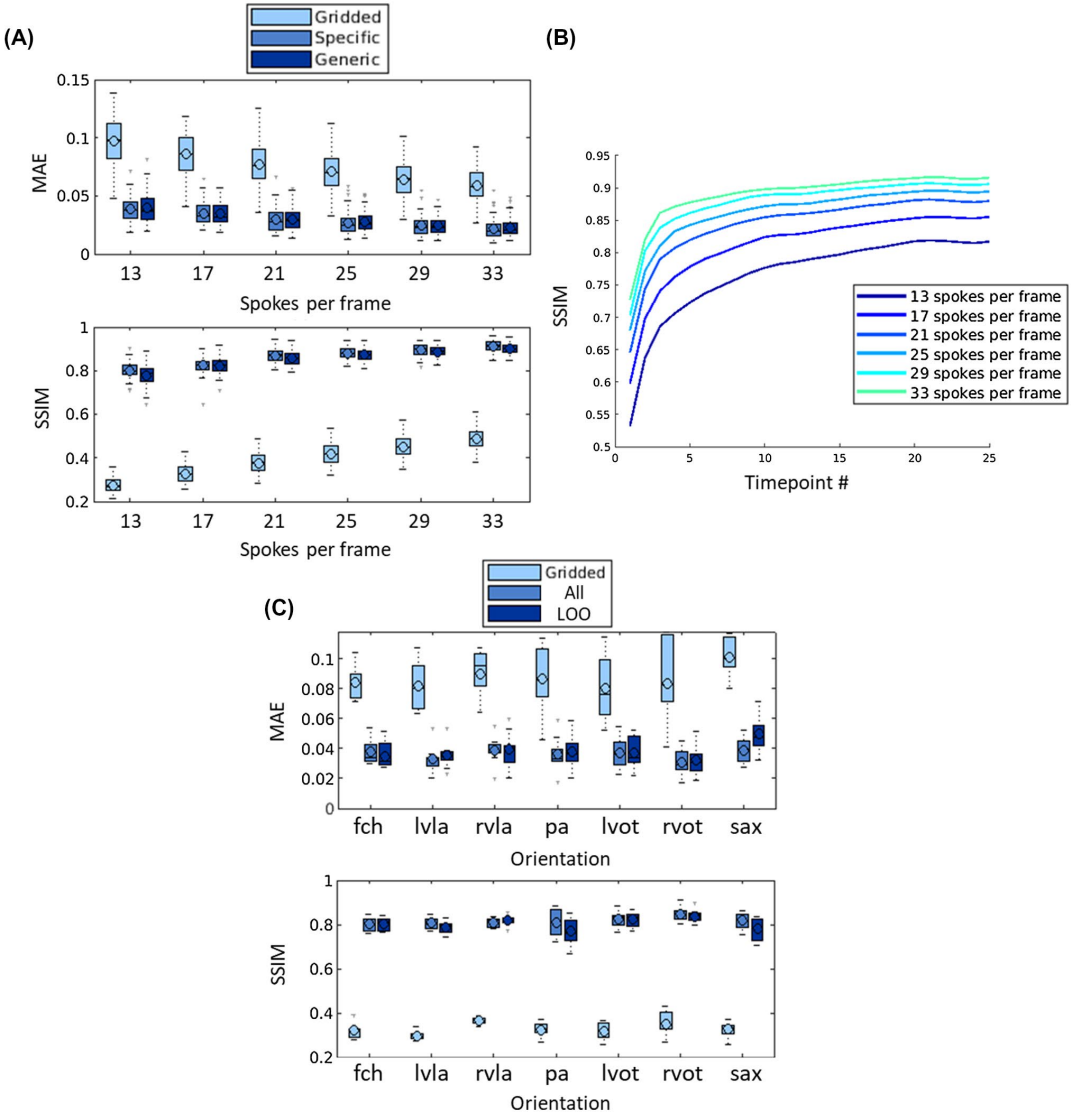
(A) Boxplots from the acceleration experiment showing MAE and SSIM over the test set data for gridded images and the reconstructions from the specific acceleration networks and generic acceleration network. (B) SSIM for the test set in the different frames within the series, showing the gradual improvement in image quality as the number of previous frames accumulate. (C) Boxplots from the orientation experiment comparing gridded reconstructions and network dealiasing, including all orientations at training or excluding the orientation being tested. MAE, mean absolute error; SSIM, structural similarity index measure

**FIGURE 5:**
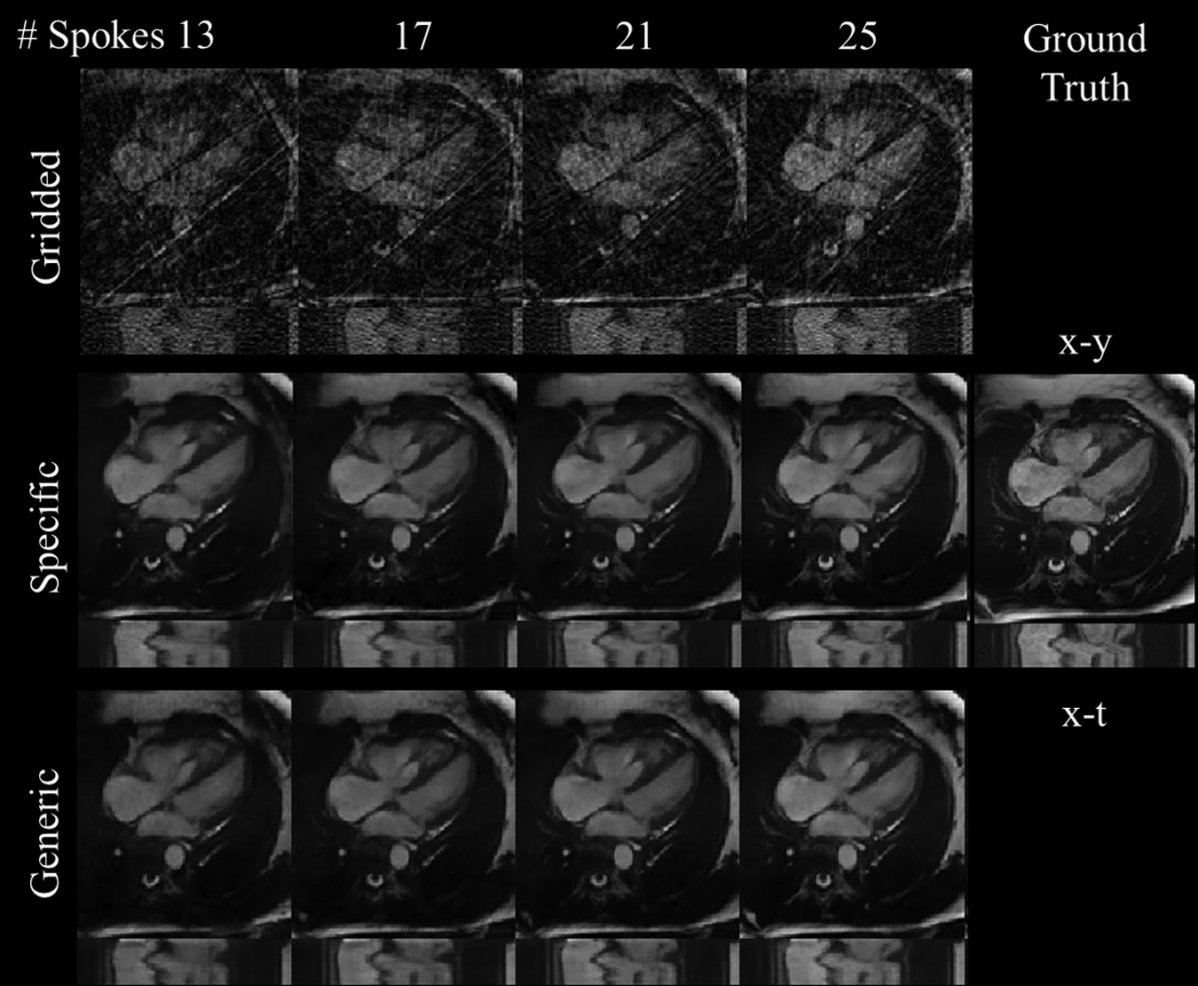
Acceleration experiment. Far right: ground truth x‐y (t = 15) and x‐t images (y = 64). Top row: simulated gridded images using 13, 17, 21, and 33 spokes. Middle row: reconstructed images obtained using networks specifically trained for the corresponding acceleration rate. Bottom row: reconstructed images obtained using the same “generic” network trained from randomly picked acceleration rates. Corresponding video including all acceleration rates can be found in Supporting Information Video S1

MAE and SSIM for the gridded images, as well as reconstructions with the specific acceleration and generic acceleration networks, are shown in Figure 4A; accompanying images are shown in Figure 5 and Supporting Information Figure S2. For all reconstructions, MAE and SSIM improved as the number of spokes per frame increased. On visual inspection, the images became less blurred as the number of spokes per frame increased. There were no obvious hallucinated features at any of the acceleration factors. The generic acceleration network performed slightly worse than specific acceleration networks at all acceleration rates for SSIM (*P* ≤ 0.01) and at 25 and 33 spokes/frame for MAE (*p* ≤ 0.01). However, the deterioration was negligible, with little visual difference and a maximum SSIM loss ≤ 0.02 between the images reconstructed using the specific and generic acceleration networks (Figure 5 and Supporting Information Video S1). All quantitative results can be seen in Supporting Information Table S1, including additional mean squared error and peak SNR metrics. The worst, median, and best test case images for the generic network in terms of SSIM (averaged over all accelerations) are shown in Supporting Information Video S2.

In Figure 4B, the frame‐by‐frame average SSIM shows how reconstructed image quality improves as more frames are reconstructed. The SSIM quickly reaches a plateau for all acceleration rates, although this took more frames at higher acceleration rates.

### 3.2 Catheter visualization

Reconstructed image series of the simulated catheter using the same generic network for all acceleration rates are shown in Supporting Information Video S3. Images successfully depicted the appearing and disappearing devices at all acceleration rates. The image fidelity and reconstructed device improves with the number of spokes per frame. In subsequent experiments, the number of radial spokes is set at 17 (ie, 54 ms per frame).

### 3.3 LOO orientation experiment

**FIGURE 6:**
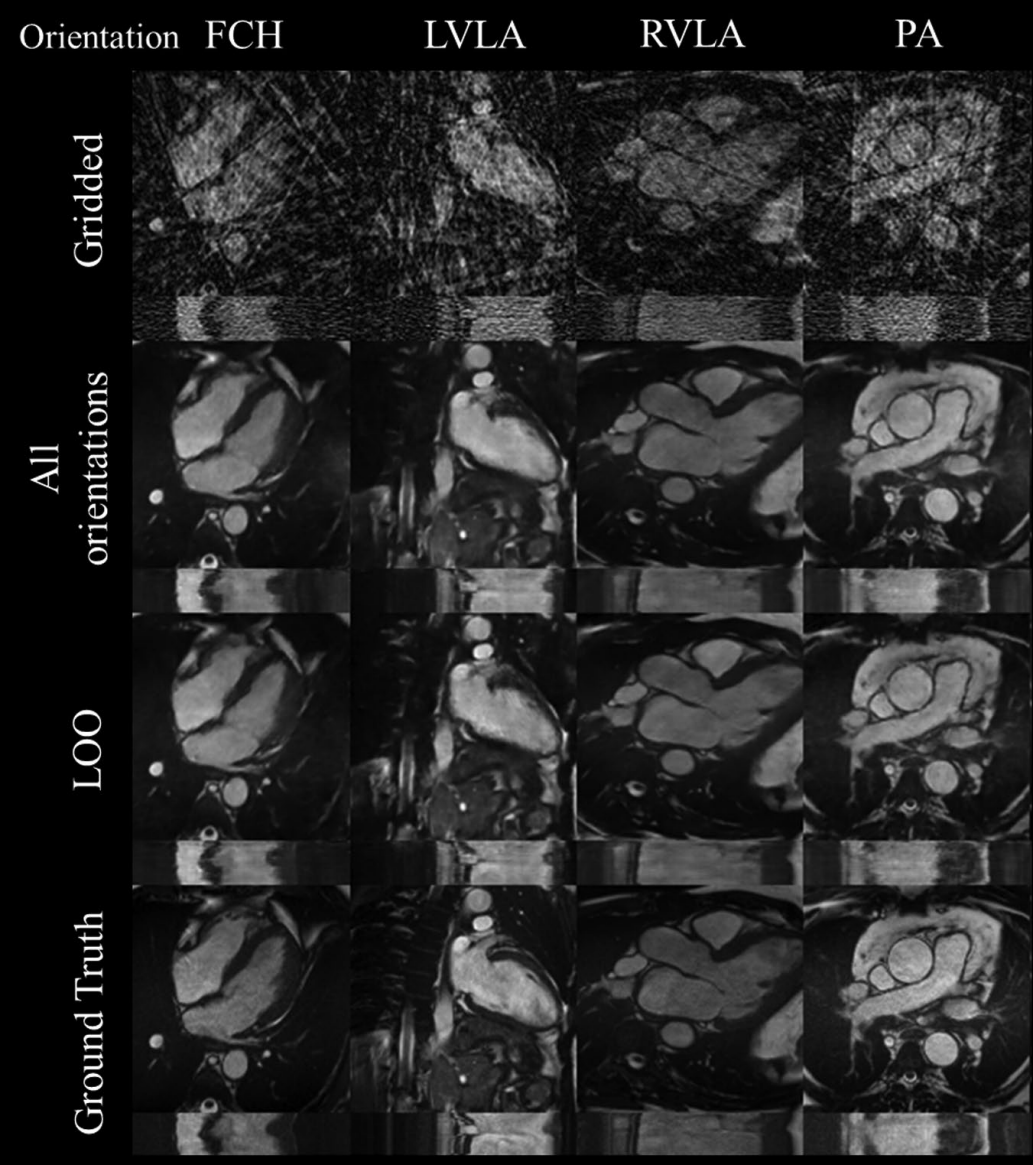
Orientation experiment. From left to right: test set x‐y (t = 15) and x‐t (y = 64) images from FCH, LVLA, RVLA, and PA orientations. Gridded images (17 spokes), images reconstructed by a network including all orientations, images reconstructed by a network that had no images taken from that particular orientation (LOO), and ground truth images are compared. Corresponding video including all orientations can be found in Supporting Information Video S4. FCH, 4 chamber; LOO, leave‐one‐out; LVLA, left ventricular long axis; PA, pulmonary artery; RVLA, right ventricular long axis

MAE and SSIM for the gridded images, as well as reconstructions using the LOO and all orientations networks for the different orientations, are shown in Figure 4C. For most orientations (4 chamber, left ventricular outflow tract, right ventricular long axis, and right ventricular outflow tract), there were no statistically significant differences in performance between the 2 networks (*P* > .05). The LOO networks did perform less well (*P* < .05) for the pulmonary artery, short axis, and left ventricular long axis orientations in terms of SSIM. However, the effect size was relatively small, as can be seen qualitatively in Figure 6 (4 of 7 orientations) and Supporting Information Figure S3 and Video S4 (for all orientations) and as can be seen quantitatively with a maximum loss in SSIM of 0.04. All quantitative results can be seen in Supporting Information Table S2, including mean squared error and peak SNR.

### 3.4 In vivo experiments

Supporting Information Video S5 shows an interactive in vivo acquisition from a healthy subject using the proposed framework. Changes of orientations were performed interactively both abruptly and through continuous motion between right ventricular outflow tract, pulmonary artery, and short axis. Transition periods can be observed before convergence to good image quality. At the start of the acquisition, image quality stabilizes quickly (approximately 100‐150 ms). However, when the orientation was changed significantly during scanning, the image appeared to “morph” between the 2 orientations over ~10 frames (~0.5 s).

When using 17 spokes per frame, the latency over 100 measurements between the Gadgetron data transmission and image visualization was measured at 39.4 ± 6.3 ms (mean ± SD). The lowest/median/highest latency measured were 31.9/37.6/81.1 ms. On average, latency included 1 ms for preprocessing, 6 ms for gridded reconstruction, 19 ms for denoising, and 13 ms of other tasks (including back‐and‐forth data transmission, scaling, and format conversions). These steps can be performed in parallel for the different frames, leading to a maximal output frame rate of ~52 frame/s (ie, 19 ms/frame). The short reconstruction and transmission times meant that there was no increase in latency during the scan when acquiring at a temporal resolution of 54 ms/frame.

The network seemed to generalize well when modifying the acquired FOVs and imaging resolutions, showing qualitatively no differences in image quality in all cases (Supporting Information Video S6).

**FIGURE 7:**
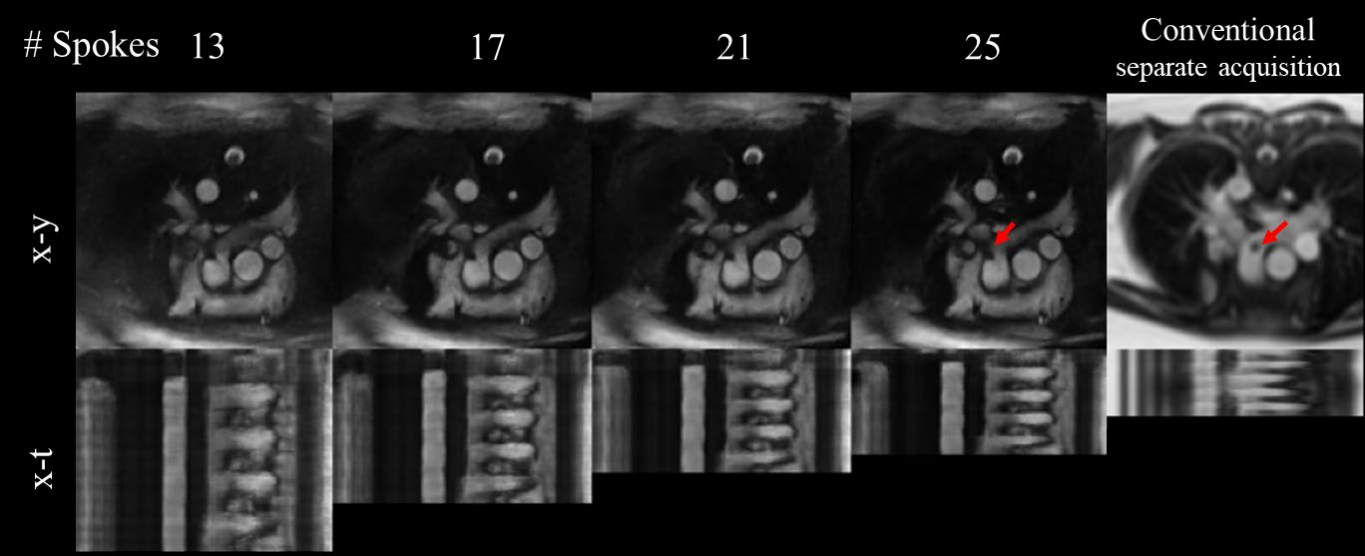
From left to right: x‐y (t≈1.36 s) and x‐t (y = 64) images of the pulmonary artery view of a catheterized patient reconstructed at 13, 17, 21, and 25 spokes per frames and corresponding images from a separate conventional real‐time Cartesian scan. The balloon is indicated with red arrows in the conventional and 33 spokes images. The x‐t frames show the first 4.16 s of acquisition. Corresponding video including all accelerations can be found in Supporting Information Video S7

Images were also acquired in 3 catheterized patients. Images of a pulmonary artery view (with catheter) reconstructed using 13, 17, 21, 25, 29, and 33 spokes per frame and compared to the conventional Cartesian real‐time acquisition are shown in Figure 7 (for 4 of 6 accelerations) and Supporting Information Figure S4 and Video S7 (for all accelerations). The balloon of the catheter was observed at all temporal resolutions, and the radial real‐time images appear visually sharper than the conventional Cartesian real‐time data.

**FIGURE 8:**
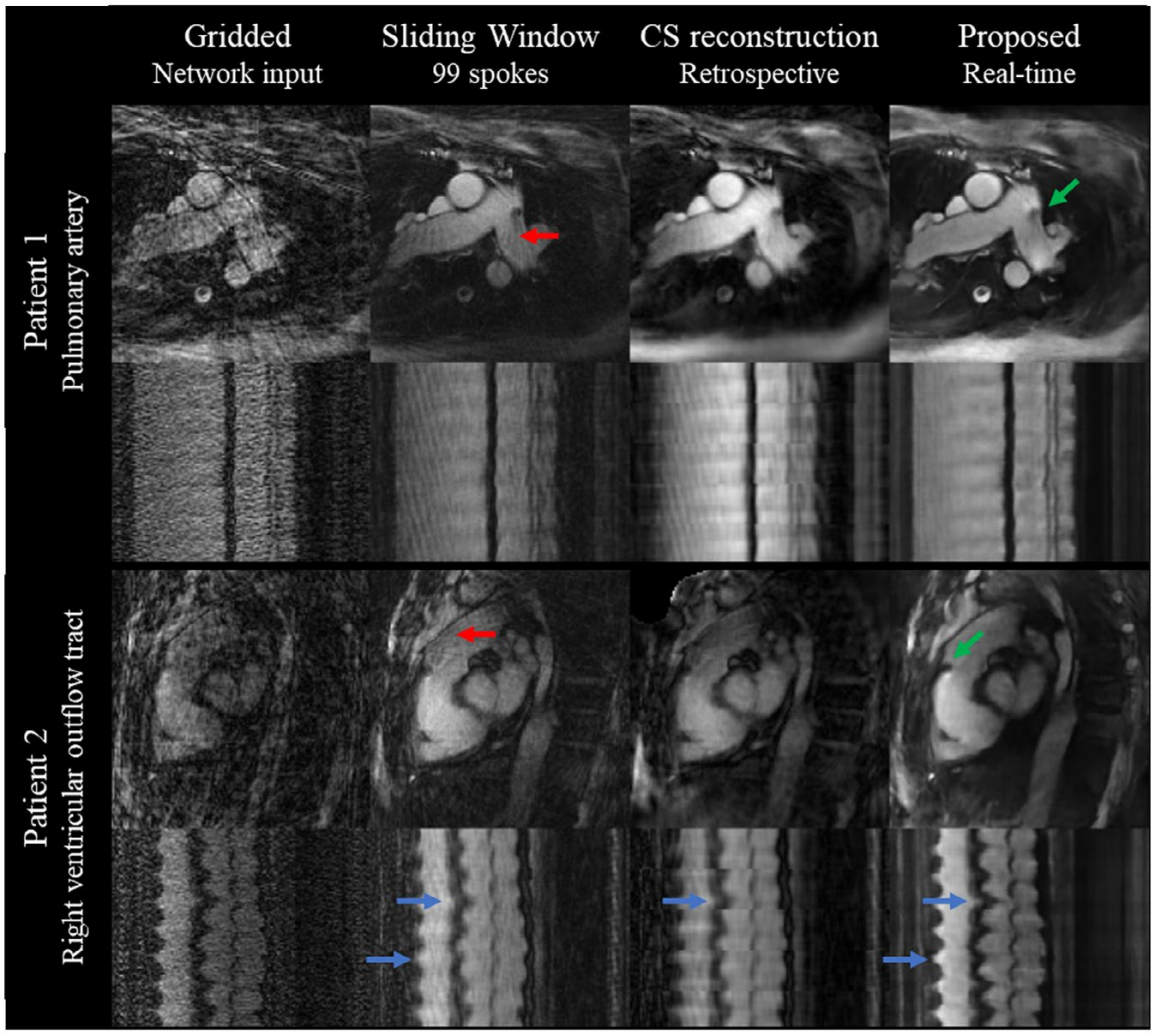
Pulmonary artery (top) and right ventricular outflow tract (bottom) views in 2 different catheterized patients. The x‐y (frame = 50) and x‐t (y = 64) frames are shown for the gridded images (input to the network), sliding window (99 spokes), retrospective CS with temporal TV regularization, and proposed real‐time DL reconstructions. The catheter is indicated by the green arrow in the proposed reconstructions. Residual streaking (red) and temporal blurring (blue arrows) are highlighted when compared to the proposed DL reconstruction. Corresponding video can be found in Supporting Information Video S8. CS, compressed sensing; TV, total cariation; DL, deep learning

Gridded, sliding window, CS, and DL reconstructed images for pulmonary artery and right ventricular outflow tract orientations are compared in Figure 8 (and Supporting Information Video S8). The catheter is seen in sliding window, CS, and DL reconstructions; however, the sliding window and CS reconstructions showed more residual aliasing artefacts and greater temporal blurring compared to the DL reconstruction. Furthermore, the sliding window required the acquisition of 41 more spokes before the start of the reconstruction, leading to an additional delay of 130 ms compared to the proposed DL reconstruction. The CS reconstruction performed on the same computer as the DL reconstruction took 2250 ms/frame, approximately ~57 times longer than the DL reconstruction of the same data, and only could be performed after the whole time series was acquired.

## 4 DISCUSSION

In this study, we demonstrated the possibility of real‐time imaging and rapid reconstruction with low latency using a multi‐scale recurrent neural network. The proposed model was tested at different acceleration rates and orientations showing good generalization properties. The full reconstruction only required a single gridding step and DL inference, making it rapid compared to state‐of‐art iterative methods. A proof‐of‐concept framework was successfully tested for interactive real‐time imaging at the scanner on patients during cardiac catheterization.

### 4.1 Network architecture

Real‐time Cardiac MR has improved significantly since the advent of iterative reconstructions such as CS, particularly when combined with non‐Cartesian sampling. However, long reconstruction times have limited translation into the clinical environment. Recently, machine‐learning reconstructions have been shown to compare favorably with CS. One example used a U‐Net to remove aliasing artefact from undersampled gridded radial real‐time images and was shown to be ~5 times faster than a comparable CS reconstruction.^14^ A benefit of this technique is that it only requires magnitude training data, which is readily available in most centers from routinely stored cardiac MRI exams. The short reconstruction times of the machine‐learning approach make it attractive for interventional Cardiac MR.

The previous study^14^ described above was based on a 3D (2D+time) convolutional neural network, which reconstructed multiple frames in batches and is therefore not suitable for interactive imaging. A simple 2D U‐Net could provide low latency visualization, but image quality has previously been shown to be poor with significant temporal jitter.^14^ Thus, in our proposed network architecture we used a 2D U‐Net with ConvLSTM layers at multiple scales. This allowed frame‐by‐frame reconstruction while still being able to exploit spatiotemporal redundancies.

### 4.2 Simulation results

A key requirement for catheter guidance is flexibility in terms of acceleration and orientation. Although there was a small difference in the image quality between specific and generic acceleration networks, this was hard to visually perceive. Thus, we feel that the generic network is suitable for catheter guidance. The theoretical benefit of using a generic network is that frame rate could be altered interactively to meet the exact requirements of the interventionalist. Differences in image quality with LOO/“all orientations” trained networks were also difficult to observe. This suggests that our network was generalizable enough to reconstruct orientations that were not in the training data (as well as catheters and guidewires). This is vital for catheter guidance because optimum visualization of a devices might not be in a conventional orientation.

One issue with using LSTM layers was that it took a few frames for image quality to stabilize. This is more apparent in the in vivo study and will be discussed in the next section.

### 4.3 In vivo study

The interactive framework was tested in a healthy subject in whom we could assess timing and latency in a controlled environment. Importantly, our framework was run on a simple midrange laptop and GPU, demonstrating that this approach did not rely on large amounts of computational resources.

Inference time for the proposed model was on average 19 ms (longest reconstruction step), with an overall latency between coil compression and image visualization of 39 ms, which is significantly lower than the acquisition time (54 ms). In the proposed framework, the different frames can be preprocessed, gridded, and artefact‐suppressed in parallel (ie, frame 2 can be zero‐filled, whereas frame 1 is being artefact‐suppressed), leading to a maximum output frame rate of ~52 frames/s (19 ms/frame), similar to other state‐of‐the‐art methods^8^, ^9^ but using only a single mid‐range GPU rather than multi‐GPUs.^7^, ^9^ At the proposed acquisition speed of 54 ms/frame, larger models with longer inference times could potentially be used.

We also showed that the proposed framework could perform deep artefact suppression of images acquired with different resolutions and FOV sizes without any further training. This is vital for use in clinical practice.

One issue with our framework is that it takes a number of frames for the image quality to stabilize. This is a property of unidirectional LSTM layers because they accumulate and maintain some memory of previous frames through the hidden state. This aids artefact suppression by leveraging spatiotemporal redundancies but does result in the “morphing” artefact seen during abrupt changes in orientation (see Supporting Information Video S5). Although the transition times are relatively short (~0.5 s), they are visually distracting and need to be remedied for general clinical use. The most obvious solution is to either reset the hidden states after large changes in orientation or train the network to start from non‐zero states (by not re‐setting states between time series at training). However, this will require some changes to our framework, particularly in the use of TensorFlow Serving.

The patient part of this study was performed to ascertain if the catheter could be visualized using our DL reconstruction, and to compare image quality to a sliding window and CS reconstruction of the same data and to conventional Cartesian real‐time imaging.

Although not present in any images in the training or validation dataset, the catheter balloon was visible in all patients using the DL reconstruction. This is in keeping with the simulation study and suggests that our method could be used with a range of MR compatible catheters. Higher temporal and spatial resolutions were reached with our DL reconstruction than with the Cartesian real‐time data. Interestingly, our DL reconstruction provided a good combination of artefact removal and temporal fidelity, with less residual artefact and temporal blurring than both the sliding window and the CS reconstructions.

Increasing regularization may have removed these artefacts in the CS reconstruction, but this would also have reduced motion fidelity. More importantly, it would not have been possible to achieve a sufficiently low latency with CS to enable catheter guidance.

### 4.4 Study limitations

The main limitation of this study was that our framework was not used to perform actual catheter guidance. This was because it is a proof‐of‐concept study, and we believe that this method should be further tested (possibly in large animal models) prior to clinical use.

Another limitation is that the current method was not tested against state‐of‐the‐art real‐time reconstructions (real‐time GRAPPA^5^ or fast nonlinear inverse reconstructions^9^) because frameworks are prohibitively complicated to implement. Comparisons of image quality and latency between techniques on the same imaging data and using the same hardware would be relevant. The current reconstruction relies on GPUs, which are not available on all commercially available scanners. Future works will potentially investigate reducing further the inference time by reducing the model size or through quantization and pruning for CPU based real‐time reconstructions enabling full integration in MR systems.

Although no ground truth is available in vivo, lower image quality of both the cardiac structures and the catheter was observed when compared to simulations. In the simulated images, the catheter balloon tip was represented by a simple Gaussian, which was either fully in or out of the slice. The actual balloon has a more complex geometry and will be partial volumed, explaining the mismatch between the simulated and actual catheter visualization. More generally, there is a mismatch between the simulated undersampled images and the acquired multi‐coil gridded images. Improving the simulation accuracy, integrating previously measured coil sensitivity maps, and further improvements to the acquisition scheme could help improve the final reconstructed images.

Finally, a possible risk with our approach is “hallucination” of features, particularly because our network architecture does not include any data consistency. In our study, we did not observe any obvious hallucinated or missing features. This is in keeping with previous studies that show that tiny golden angle radial sampling enables accurate reconstruction, even as a postprocessing step.^14^ Nevertheless, further optimizations (use of data consistency terms, unrolled optimizations,^11^, ^12^ and complex valued networks^32^, ^33^) might increase reconstruction accuracy and further improve image quality.^17^, ^32^ However, these will be heavily constrained by reconstruction times and the lack of large amounts of raw k‐space data for training. Other approaches could be investigated, such as including an additional loss in k‐space to penalize data inconsistency while keeping reconstruction times short. Furthermore, these rely on complex image/raw k‐space data for training of the networks, which is less available than the DICOM magnitude data used for training in this study.

## 5 CONCLUSION

Deep artifact suppression was successfully demonstrated in the time critical application of non‐Cartesian real‐time interventional cardiac MR, showing promising performance in terms of both image quality and reconstruction times when compared to CS reconstructions of the same raw data.

## Supporting information


**FIGURE S1** SSIM through time corresponding to images of the same data reconstructed without warm up, with random Gaussian noise warm up and with array of zeros warm up. Warm up with arrays of zeros affects the SSIM more durably while Gaussian noise quickly reaches a similar SSIM to the framework without warm up while reducing initial latency
**FIGURE S2** Acceleration Experiment. Far right: Ground truth x‐y (t = 15) and x‐t images (y = 64). Top row: Simulated gridded images using 13, 17, 21, 25, 29 and 33 spokes. Middle row: Reconstructed images obtained using networks specifically trained for the corresponding acceleration rate. Bottom row: Reconstructed images obtained using the same “generic” network trained from randomly picked acceleration rates. Corresponding video can be found in Supporting Information Video S1
**FIGURE S3** Orientation Experiment. From left to right: Test set x‐y (t = 15) and x‐t (y = 64) images from four chambers (FCH), left ventricular long axis (LVLA), right ventricular long axis (RVLA), pulmonary artery (PA), left ventricular outflow tract (LVOT), right ventricular outflow tract (RVOT) and short axis (SA) orientations. Gridded images (17 spokes), images reconstructed by a network including all orientations, images reconstructed by a network which had no images taken from that particular orientation (LOO), and ground truth images are compared. Corresponding video can be found in Supporting Information Video S4
**FIGURE S4** From left to right: x‐y (t≈1.36 seconds) and x‐t (y = 64) images of the pulmonary artery view of a catheterized patient reconstructed at 13, 17, 21, 25, 29 and 33 spokes per frames and corresponding images from a separate conventional real‐time Cartesian scan. The balloon is indicated with red arrows in the conventional and 33 spokes images. The x‐t frame show the first 4.16 seconds of acquisition. Corresponding video including all accelerations can be found in Supporting Information Video S7
**TABLE S1** Acceleration Experiment. The mean absolute error (MAE), mean squared error (MSE), peak signal‐to‐noise ratio (PSNR) and structural similarity index measure (SSIM) reported for the different reconstructions. Average differences and Wilcoxon rank test p‐value to assess differences between specific and generic deep artifact suppression are reported
**TABLE S2** Orientation Experiment. The mean absolute error (MAE), mean squared error (MSE), peak signal‐to‐noise ratio (PSNR) and structural similarity index measure (SSIM) reported for various orientations for gridded images and reconstructed images using a network which has seen all orientations and one which has seen all but the tested orientation (“LOO”). Bias and Wilcoxon rank test p‐value to assess differences between seen and unseen denoising are reported

**VIDEO S1** Acceleration Experiment. Far right: Ground truth images. Top row: Simulated gridded images using 13, 17, 21, 25, 29 and 33 spokes. Middle row: Reconstructed images obtained using networks specifically trained for the corresponding acceleration rate. Bottom row: Reconstructed images obtained using the same “generic” network trained from randomly picked acceleration rates

**VIDEO S2** Acceleration Experiment. From top to bottom: Test set images exhibiting the worst, median and best measured SSIM (averaged over all accelerations). From left to right: Gridded (13 spokes), denoised (13, 17, 21, 25, 29, 33 spokes) and Ground Truth images

**VIDEO S3** Catheter Visualization. Top row: Simulated catheter balloon images reconstructed at 13, 17, 21, 25, 29 and 33 spokes per frame using the “generic” network and ground truth images. Bottom row: Corresponding images with a simulated guidewire

**VIDEO S4** Orientation Experiment. From left to right: Test set videos from four chambers (FCH), left ventricular long axis (LVLA), right ventricular long axis (RVLA), pulmonary artery (PA), left ventricular outflow tract (LVOT), right ventricular outflow tract (RVOT) and short axis (SA) orientations. Gridded images (17 spokes), images reconstructed by a network including all orientations, images reconstructed by a network which had no images taken from that particular orientation (LOO), and ground truth images are compared

**VIDEO S5** Proof of concept interactive acquisition in a healthy subject. Changes of orientations are performed interactively both abruptly and through continuous motion between RVOT, PA and SAX. Short transition periods can be observed before convergence to good image quality

**VIDEO S6** Separate acquisitions of the same pulmonary artery view with (top) varying acquired field of views (FOV) of 320 × 320 mm^2^, 360 × 360 mm^2^, 420 × 420 mm^2^ (fixed resolution: 1.67 × 1.67 mm^2^) and (bottom) resolutions of 2 × 2 mm^2^, 1.67 × 1.67 mm^2^ and 1.5 × 1.5 mm^2^ (fixed FOV: 320 × 320 mm^2^)

**VIDEO S7** From left to right: Pulmonary artery view of a catheterized patient reconstructed at 13, 17, 21, 25, 29 and 33 spokes per frames and corresponding images from a separate conventional real‐time Cartesian scan. The images are shown with the different framerates corresponding to the acquisition framerate

**VIDEO S8** Pulmonary artery (top) and right ventricular outflow tract (bottom) views in two different catheterized patients. The images are shown for the gridded images (17 spokes/frame), sliding window (99 spokes/frame), retrospective compressed sensing (CS with temporal TV regularization) and proposed real time ML reconstructions
