# Supplementary material for: Real‐time deep artifact suppression using recurrent U‐Nets for low‐latency cardiac MRI
Source: Magn Reson Med. 2021 May 25;86(4):1904–16. doi: 10.1002/mrm.28834 (PMC8613539; doi:10.1002/mrm.28834)
Supplement: Supplementary file 1 — FIGURE S1 SSIM through time corresponding to images of the same data reconstructed without warm up, with random Gaussian noise warm up and with array of zeros warm up. Warm up with arrays of zeros affects the SSIM more durably while Gaussian noise quickly reaches a similar SSIM to the framework without warm up while reducing initial latency FIGURE S2 Acceleration Experiment. Far right: Ground truth x‐y (t = 15) and x‐t images (y = 64). Top row: Simulated gridded images using 13, 17, 21, 25, 29 and 33 spokes. Middle row: Reconstructed images obtained using networks specifically trained for the corresponding acceleration rate. Bottom row: Reconstructed images obtained using the same “generic” network trained from randomly picked acceleration rates. Corresponding video can be found in Supporting Information Video S1 FIGURE S3 Orientation Experiment. From left to right: Test set x‐y (t = 15) and x‐t (y = 64) images from four chambers (FCH), left ventricular long axis (LVLA), right ventricular long axis (RVLA), pulmonary artery (PA), left ventricular outflow tract (LVOT), right ventricular outflow tract (RVOT) and short axis (SA) orientations. Gridded images (17 spokes), images reconstructed by a network including all orientations, images reconstructed by a network which had no images taken from that particular orientation (LOO), and ground truth images are compared. Corresponding video can be found in Supporting Information Video S4 FIGURE S4 From left to right: x‐y (t≈1.36 seconds) and x‐t (y = 64) images of the pulmonary artery view of a catheterized patient reconstructed at 13, 17, 21, 25, 29 and 33 spokes per frames and corresponding images from a separate conventional real‐time Cartesian scan. The balloon is indicated with red arrows in the conventional and 33 spokes images. The x‐t frame show the first 4.16 seconds of acquisition. Corresponding video including all accelerations can be found in Supporting Information Video S7 TABLE S1 Acceleration Experiment. The m [file MRM-86-1904-s005.docx]

Real-time deep artifact suppression using recurrent U-Nets for low latency Cardiac Magnetic Resonance imaging: Supporting Information

*Supporting Information Text S1: On-site reconstruction implementation for in vivo experiments.*

On site reconstructions were performed using the Gadgetron framework. The ‘generic’ model was pre-loaded and warmed up on the external computer using the TensorFlow Serving API. During acquisition, the data is first coil compressed (8 virtual coils) based on pre-scan information (vendor software), the Gadgetron framework then converts the coil compressed data to ISMRM Raw Data format (ISMRMRD, a vendor neutral file format (1)) and sends it over to the external computer via TCP/IP where preprocessing, gridding and coil combination is performed. Using Gadgetron’s foreign language interface, single coil 2D images are forwarded to a Python module (client) for ML based denoising. In Python, a light gRPC (Remote Procedure Call) client was used to efficiently convert data as Protocol Buffers and send requests from the Gadgetron to the TensorFlow server. 30% of the GPU memory was preallocated for the TensorFlow server for inference and 70% for the rest (Gadgetron-based image preprocessing -i.e. trajectory and density compensation weights calculations-, gridding and Fourier Transforms). The reconstructed 2D images are then sent back to the scanner as soon as they are reconstructed for visualization.

1. Inati SJ, Naegele JD, Zwart NR, et al. ISMRM Raw data format: A proposed standard for MRI raw datasets. Magn. Reson. Med. 2017;77:411–421.

*Supporting Information Figure S1:*


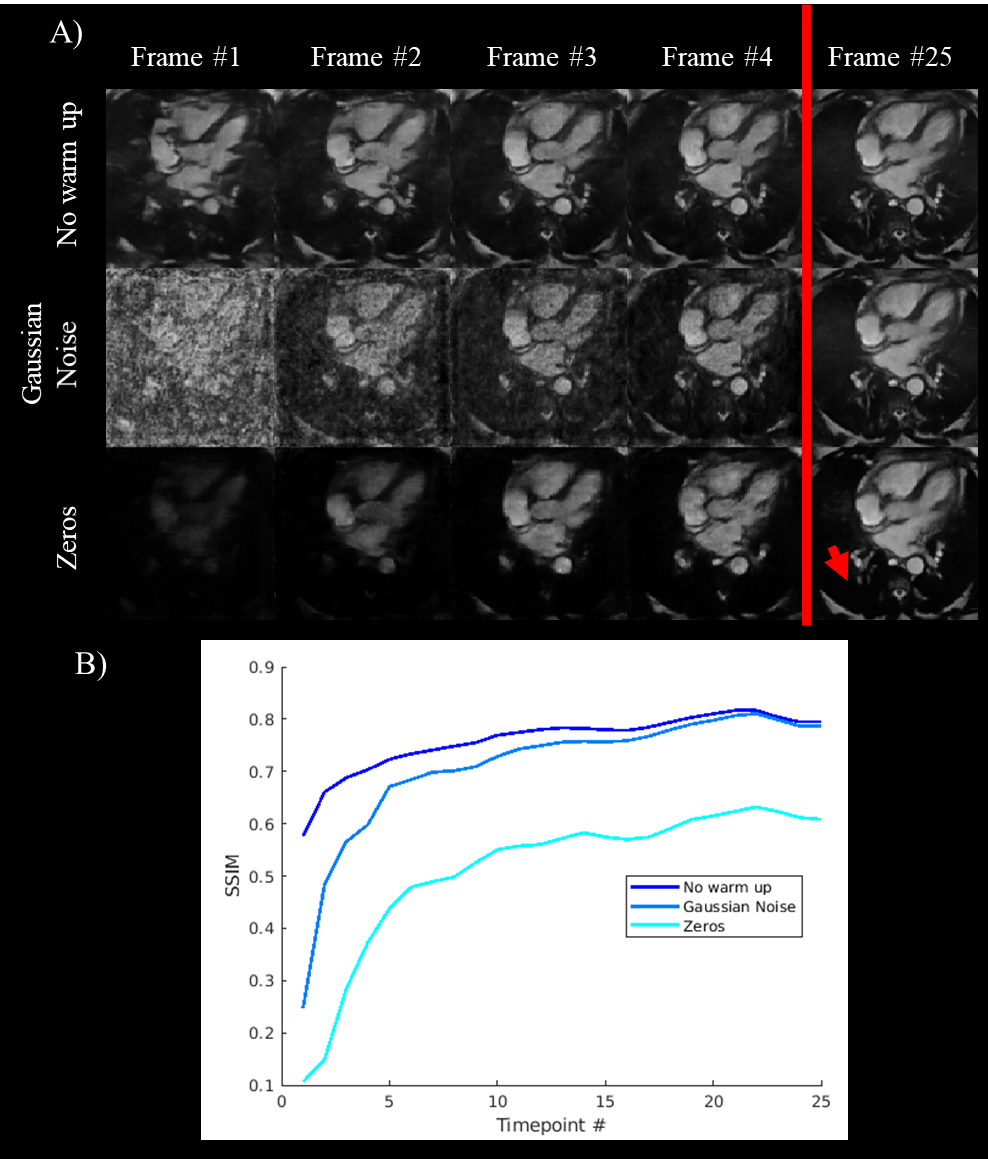


Supporting Information Figure S1. SSIM through time corresponding to images of the same data reconstructed without warm up, with random Gaussian noise warm up and with array of zeros warm up. Warm up with arrays of zeros affects the SSIM more durably while Gaussian noise quickly reaches a similar SSIM to the framework without warm up while reducing initial latency.

*Supporting Information Figure S2:*


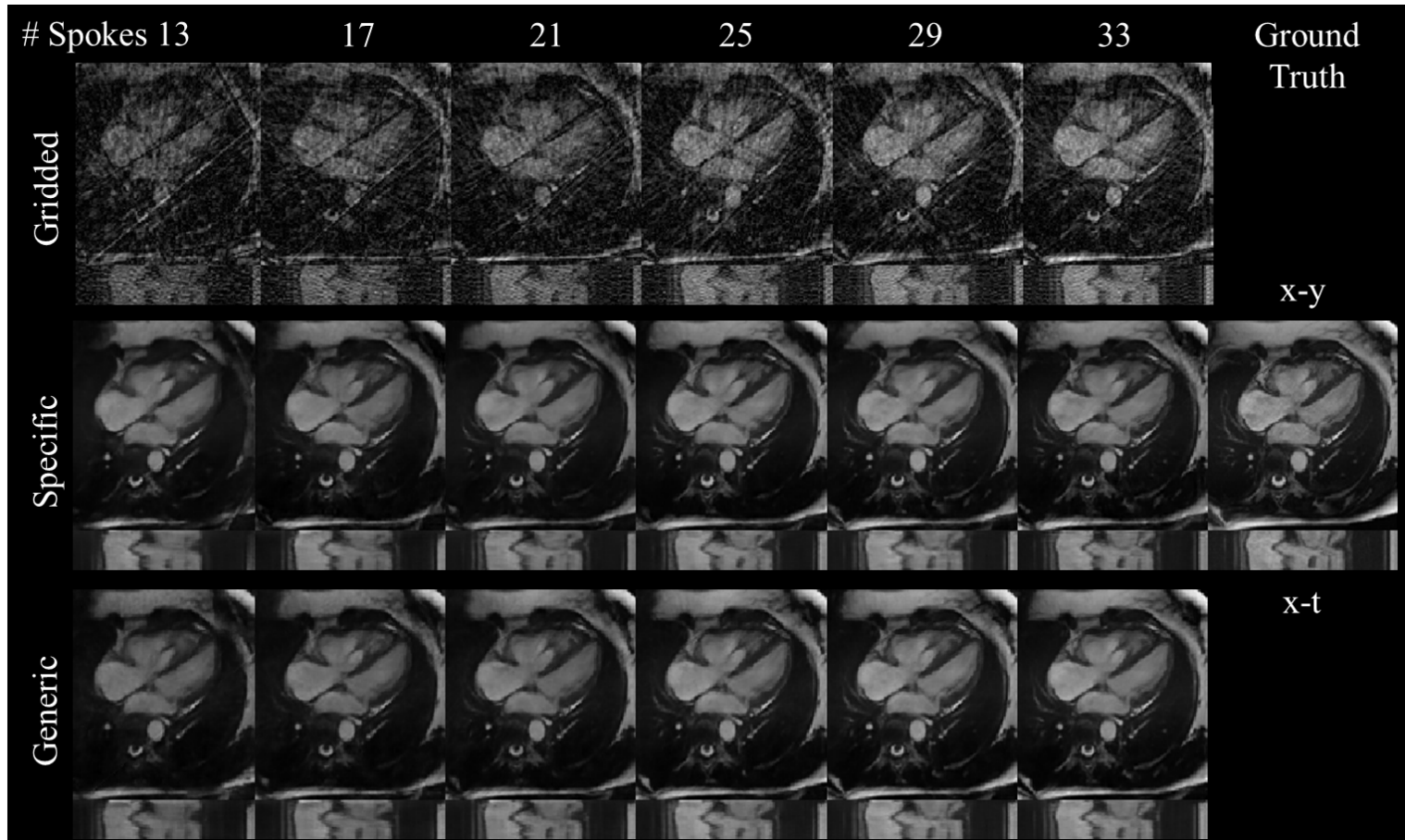


Supporting Information Figure S1. Acceleration Experiment. Far right: Ground truth x-y (t=15) and x-t images (y=64). Top row: Simulated gridded images using 13, 17, 21, 25, 29 and 33 spokes. Middle row: Reconstructed images obtained using networks specifically trained for the corresponding acceleration rate. Bottom row: Reconstructed images obtained using the same ‘generic’ network trained from randomly picked acceleration rates. Corresponding video can be found in Supporting Information Video S1.

*Supporting Information Table S1*:
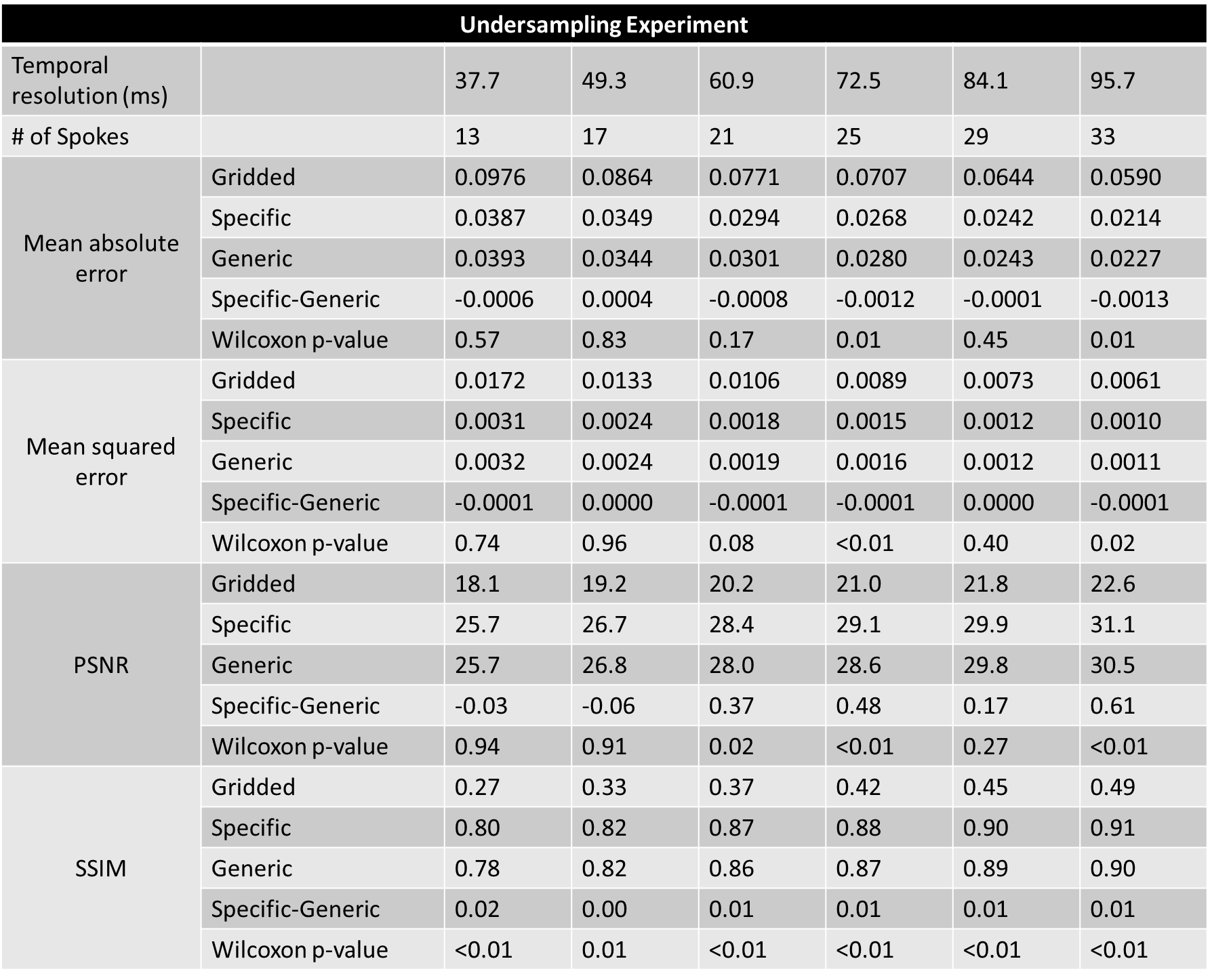


Supporting Information Table S1. Acceleration Experiment. The mean absolute error (MAE), mean squared error (MSE), peak signal-to-noise ratio (PSNR) and structural similarity index measure (SSIM) reported for the different reconstructions. Average differences and Wilcoxon rank test p-value to assess differences between specific and generic deep artifact suppression are reported.

*Supporting Information Figure S3:*


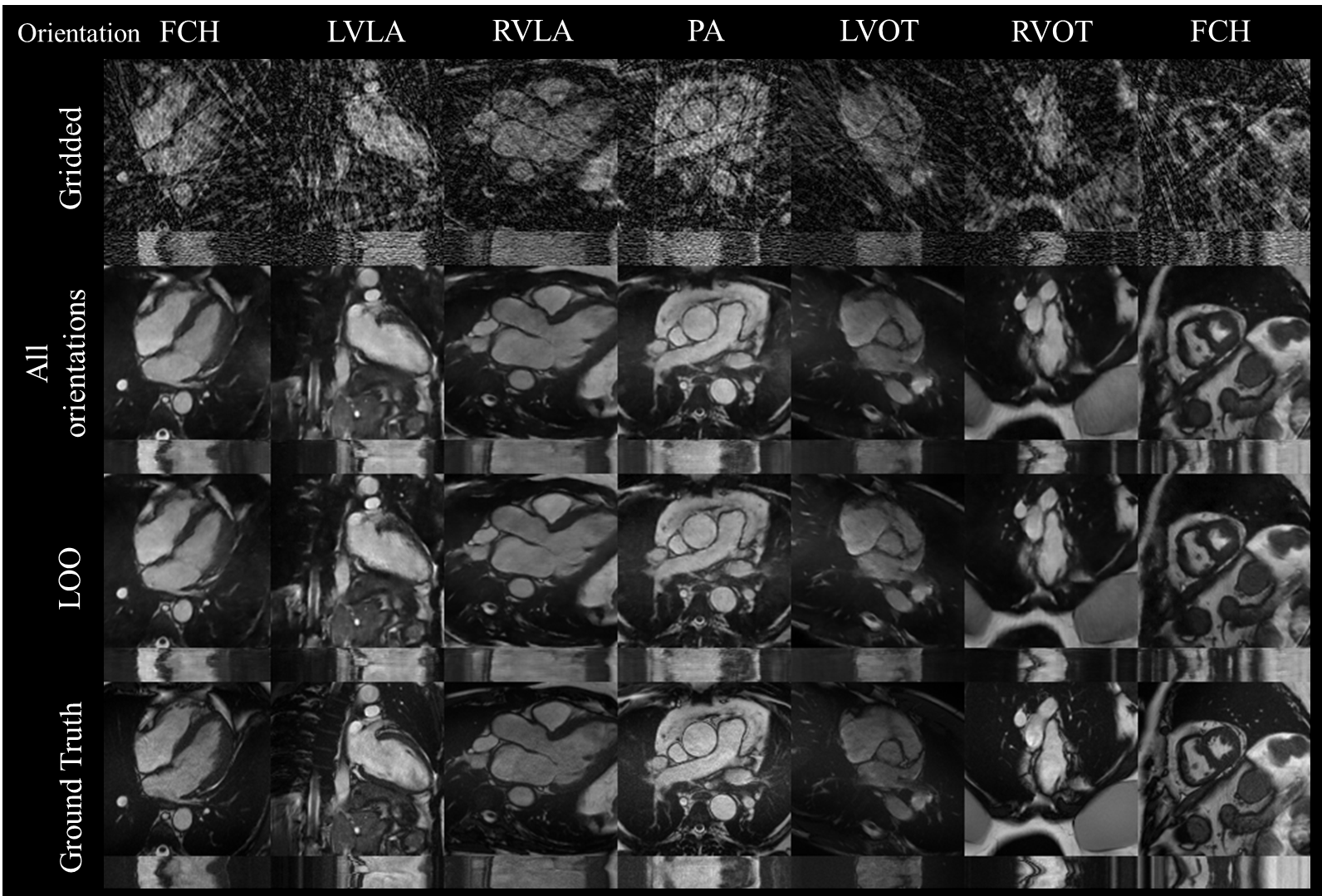


Supporting Information Figure S2. Orientation Experiment. From left to right: Test set x-y (t=15) and x-t (y=64) images from four chambers (FCH), left ventricular long axis (LVLA), right ventricular long axis (RVLA), pulmonary artery (PA), left ventricular outflow tract (LVOT), right ventricular outflow tract (RVOT) and short axis (SA) orientations. Gridded images (17 spokes), images reconstructed by a network including all orientations, images reconstructed by a network which had no images taken from that particular orientation (LOO), and ground truth images are compared. Corresponding video can be found in Supporting Information Video S4.

*Supporting Information Table S2:*


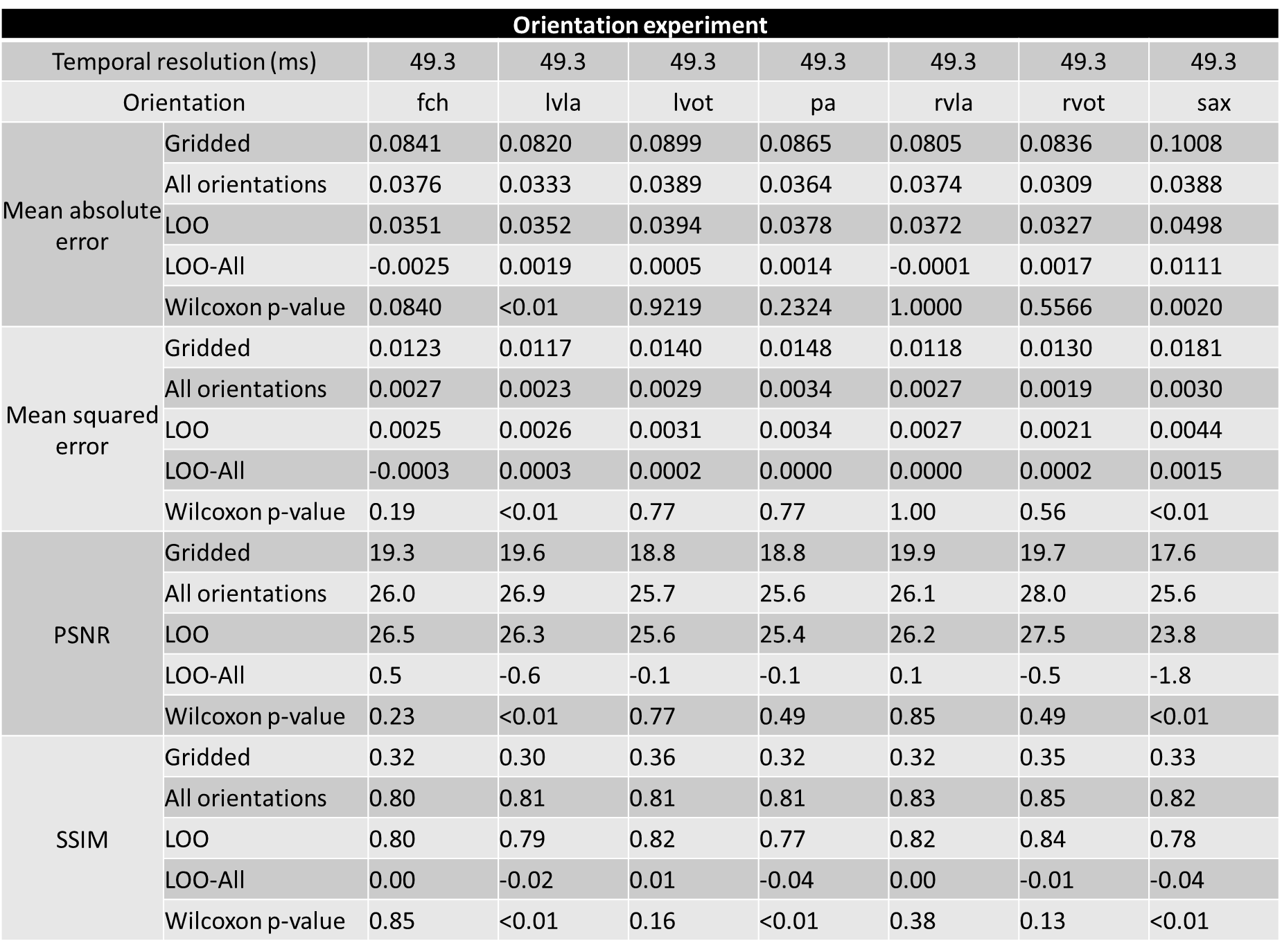


Supporting Information Table S2. Orientation Experiment. The mean absolute error (MAE), mean squared error (MSE), peak signal-to-noise ratio (PSNR) and structural similarity index measure (SSIM) reported for various orientations for gridded images and reconstructed images using a network which has seen all orientations and one which has seen all but the tested orientation (‘LOO’). Bias and Wilcoxon rank test p-value to assess differences between seen and unseen denoising are reported.

*Supporting Information Figure S4:*
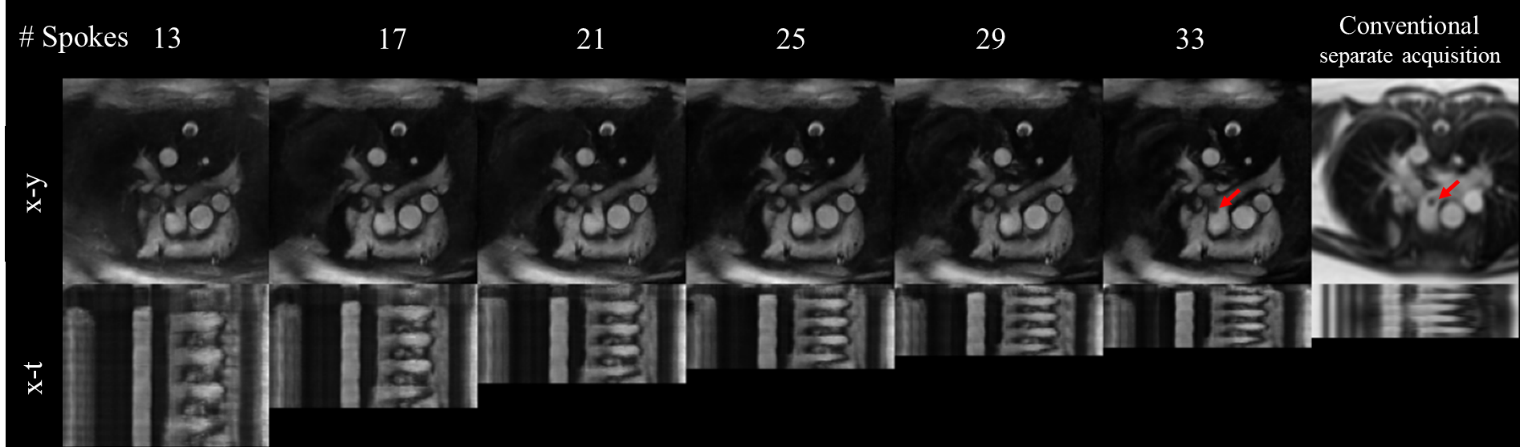


Supporting Information Figure S3. From left to right: x-y (t≈1.36 seconds) and x-t (y=64) images of the pulmonary artery view of a catheterized patient reconstructed at 13, 17, 21, 25, 29 and 33 spokes per frames and corresponding images from a separate conventional real-time Cartesian scan. The balloon is indicated with red arrows in the conventional and 33 spokes images. The x-t frame show the first 4.16 seconds of acquisition. Corresponding video including all accelerations can be found in Supporting Information Video S7.
